# Nomogram for predicted probability of cervical cancer and its precursor lesions using miRNA in cervical mucus, HPV genotype and age

**DOI:** 10.1038/s41598-022-19722-3

**Published:** 2022-09-28

**Authors:** Kiriko Kotani, Aya Iwata, Iwao Kukimoto, Eiji Nishio, Takeji Mitani, Tetsuya Tsukamoto, Ryoko Ichikawa, Hiroyuki Nomura, Takuma Fujii

**Affiliations:** 1 Department of Obstetrics and Gynecology, School of Medicine, Fujita Health University, 1-98Dengakugakubo, Toyoake, Aichi 470-1192 Japan; 2 Pathogen Genomics Center, National Institute of Infectious Diseases, 4-7-1, Gakuen, Tokyo, Musashi-murayama 208-0011 Japan; 3 Department of Pathology, School of Medicine, Fujita Health University, 1-98Dengakugakubo, Toyoake, Aichi 470-1192 Japan

**Keywords:** Cancer screening, Microbiology techniques

## Abstract

Cervical cancer is the fourth most common cancer in women worldwide. Although cytology or HPV testing is available for screening, these techniques have their drawbacks and optimal screening methods are still being developed. Here, we sought to determine whether aberrant expression of miRNAs in cervical mucus could be an ancillary test for cervical neoplasms. The presence of miRNAs in 583 and 126 patients (validation and external cohorts) was determined by real-time RT-PCR. Performance of a combination with five miRNAs (miR-126-3p, -451a -144-3p, -20b-5p and -155-5p) was estimated by ROC curve analysis. Predicted probability (PP) was estimated by nomograms comprising -ΔCt values of the miRNAs, HPV genotype and age. A combination of five miRNAs showed a maximum AUC of 0.956 (95% CI: 0.933–0.980) for discriminating cancer. Low PP scores were associated with good prognosis over the 2-year observation period (*p* < 0.05). Accuracy for identifying cancer and cervical intraepithelial neoplasia (CIN) 3 + by nomogram was 0.983 and 0.966, respectively. PP was constant with different storage conditions of materials. We conclude that nomograms using miRNAs in mucus, HPV genotype and age could be useful as ancillary screening tests for cervical neoplasia.

## Introduction

Cervical cancer is the fourth most common cancer in women, with an estimated 604,127 new cases and 341,831 deaths worldwide in 2020^1^. Persistent infection with a subset of human papillomaviruses (HPV), designated “high-risk” HPV, is a necessary cause of cervical cancer development. Despite the fact that organized cytology screening has greatly contributed to decreasing cervical cancer incidence in developed countries, cytology is not necessarily an ideal tool for screening due to its low sensitivity for detection of high-grade cervical intraepithelial neoplasia (CIN) lesions^2^. Recently, HPV DNA tests have been introduced into the screening system because these exhibit high sensitivity compared to cytology. However, their specificity is inferior because most HPV infections are transient^3^. Although HPV testing together with cytology triage remains the mainstay of screening, novel strategies under development include assessing DNA methylation, p16/Ki67 immunostaining, and the incorporation of machine learning strategies were discussed to identify cervical abnormalities^4,5^.

MicroRNAs (miRNAs), non-coding RNAs 19–25 nucleotides in length, modulate gene expression by partially pairing with the 3’ untranslated region of their target messenger RNAs^6^. About two-thirds of human messenger RNAs are thought to be regulated in this manner^7^ and approximately 2500 human miRNAs are currently recorded in the miRBase database (www.mirbase.org). Certain miRNAs have been reported to act as oncogenes or tumour suppressors^6^; in this context, aberrant expression of miRNAs in cervical cancer and its precursor lesions were previously reported^8–11^. The determination of miRNA profiles could thus be promising as an ancillary test in cervical cancer screening^11–14^. We previously reported the identification of miRNAs in the cervical mucus as a diagnostic marker for cervical neoplasia^15^. One advantage of using specimens of cervical mucus is the lower likelihood of causing any harm during the procedure. The aim of the present study was to assess the performance of a combination of miRNAs included in a nomogram for predicting the risk of cervical neoplasia. Additionally, we wished to ascertain the stability of miRNA in cervical mucus kept overnight at room temperature if not immediately frozen, as to the best of our knowledge this practically important issue had not been investigated. Therefore, we investigated whether storage temperature conditions affected the probability predicted by the nomogram.

## Results

### Identification of a novel internal reference control in cervical mucus

Because internal reference controls are required as samples and PCR stability indicators, we selected six highly expressed miRNAs showing a fold-difference < 0.1 and equivalence between disease groups including CIN1, CIN3, squamous cell carcinoma (SCC), adenocarcinoma (AD) and normal controls, as shown by microarray analysis in Supplementary Figure S1. We could not analyze miR-6089 and -6125 due to high GC-content. Real-time RT-PCR was performed to further evaluate the patterns of expression of the four candidate reference miRNAs identified by microarray analysis. Neither miR-4730 nor -miR-4327 could be amplified in only 70% of the samples, and therefore these miRNAs were excluded from further analysis. We compared the mean Ct values of the candidate internal reference control obtained from normal and cervical neoplasia groups (Supplementary Table S1). We found no significant differences in the expression of miR-3180 (Mann–Whitney U test *p* = 0.910) and miR-7109-5p (*p* = 0.383) across normal and cervical neoplasia groups. This indicates their stable levels in health and disease, and their potential for acting as endogenous controls in cervical mucus assays. RNU48 was more abundant in patients with cervical neoplasia (*p* = 0.012) than in controls, suggesting that it might introduce a bias if adopted as the normal reference. We further analyzed the stability of each candidate internal reference control using the NormFinder algorithm. We found that among three candidates, miR-3180 was the most stably expressed (stability value 0.075), followed by miR-7109-5p (0.116) and RNU48 (0.209). Combining these with miR-3180 and miR-7109-5p further reduced the NormFinder stability value to 0.069. Thus, we decided to use the average of the Ct of miR-3180 and miR-7109-5p combined as the internal reference control in the following assessments.

### Associations between miRNA levels and cervical neoplasia

**Figure 1:**
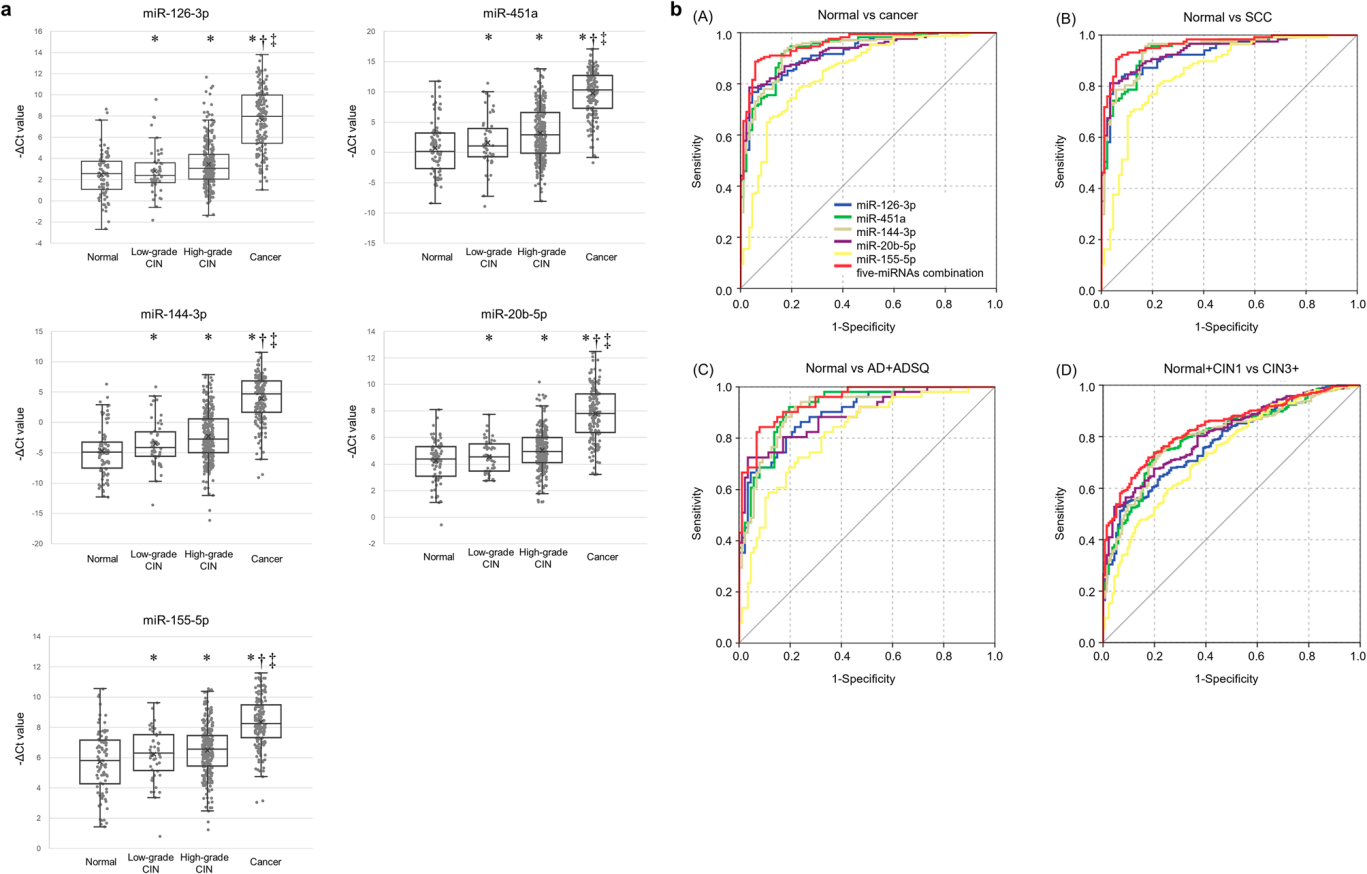
(**a**) Levels of five miRNAs by real-time RT-PCR correlate with histology. Relative expression of miRNAs was adjusted by −ΔCt values (Ct average of miR-3180 and miR-7109-5p—Ct Target miRNA). Higher -ΔCt values indicate a higher level of the miRNAs. The "x" indicates the mean value. Statistical analysis by Mann–Whitney U tests with Bonferroni correction: *p < 0.05 versus normal, †*p* < 0.05 versus Low-grade CIN: ‡*p* < 0.05 versus High-grade CIN: CIN2, CIN3 and adenocarcinoma in situ. (see “Methods” and “Results” section for more detail on the statistical analysis), (**b**) Diagnostic value of the combination of miR-126-3p, -451a, -144-3p, -20b-5p, -155-5p adjusted by the internal control, and the 5 miRNAs in combination. Receiver operating characteristics (ROC) analyses was used for discrimination of cervical neoplasia. (**A**) Normal versus cancer (**B**) Normal versus SCC (**C**) Normal versus AD + ADSQ (**D**) Normal + CIN1 versus CIN3 + . Logistic regression with 5 miRNAs and predicted probability were used as variables in the ROC procedure. The optimal cut-off value, sensitivity and specificity were determined by calculating the Youden index with respect to distinguishing patients with cervical disease. SCC: squamous cell carcinoma, ADSQ: adenosquamous carcinoma, AD: adenocarcinoma. CIN3 + : CIN3 and worse.

To identify miRNAs that are up-regulated in cervical neoplasia relative to normal cervix, we focused on the 4 miRNAs (miR-126-3p, -451a, -144-3p and -20b-5p) that were previously reported to be significantly up-regulated in SCC and AD^15^. Our previous work in the discovery cohort had shown that fold-change (disease/normal) of miR-144-3p, -451a,-126-3p, 20b-5p and -155-5p was 219.4, 369.3, 23.4, 8.3 and 8.1 for SCC, and 74.1, 65.6, 9.7, 4.3 and 2.4 for AD, respectively^15^. Here we added miR-155-5p because its fold-change was almost equivalent to that of miR-20b-5p. This was also the reason that miR-155-5p had been selected as a candidate biomarker for cervical cancer screening by others^16–18^. We explored associations of levels of the 5 miRNAs adjusted by the internal control with histology. Scatter plots and box plots showing the relative expression of miRNAs according to the -ΔCt values for each specimen are depicted in Fig. 1a. The levels of these miRNAs significantly increased with disease severity in the validation cohort as determined by the Jonckheere–Terpstra trend test. There was a significant difference across disease categories as determined by the Kruskal–Wallis test and in each category for all 5 miRNAs by the Mann–Whitney U test with Bonferroni correction. Thus, we confirmed that levels of miR-126-3p, -451a, -144-3p, -20b-5p and -155-5p in patients with CIN and cervical cancer were significantly higher than in normal controls.

### Clinical utility of assessing miRNA levels for the detection of cervical neoplasia

To explore the diagnostic value of the combination of the 5 miRNAs miR-126-3p, -451a, -144-3p, -20b-5p and -155-5p, we examined their expression in specimens from patients with cervical cancer (N = 168) and normal controls (N = 87). The Akaike Information Criterion (AIC) was used to determine which factors should be enrolled in the final model (Supplementary Table S2). In all combinations, the inclusion of the 5 miRNAs miR-126-3p, -451a -144-3p, -20b-5p and -155-5p yielded the lowest AIC value (136.9). Next, we calculated the predicted probabilities from logistic regression to obtain receiver operating characteristic (ROC) curves for combined miRNAs in Fig. 1b. The 5 miRNA-combination showed the largest AUC of 0.956 (95% confidence interval (CI), 0.933–0.980) in distinguishing patients with cervical cancer from controls. Combinations of the 4 miRNAs miR-126-3p, -451a, -20b-5p and -155-5p yielded the same AUC (0.956, 95% CI 0.933–0.979) as all 5, but the second best of AIC value (137.4), as shown in Supplementary Table S4. To further evaluate usefulness for clinical application, the cut-off points to detect cervical neoplasia were determined by the Youden index from ROC curves for the 5 miRNA-combination (Table 1). In SCC vs normal, the value for the AUC was 0.963 (0.940–0.987) with 0.91 sensitivity and 0.94 specificity. The positive likelihood ratio was 15.76 and the negative likelihood ratio was 0.10. Although the accuracy was inferior with all cancers including adenocarcinoma, adenosquamous carcinoma and squamous cell carcinoma relative to squamous cell carcinoma alone, the AUC value (0.956; CI: 0.933–0.980) and the accuracy (0.91) were acceptable for screening purposes. Additionally, we determined the accuracy for detecting CIN3 and worse (CIN3 +) lesions including CIN3 and overt cancer compared with CIN1 in addition to the normal controls. The AUC for CIN3 + was 0.836 (0.799–0.873) and the accuracy was 0.75. The clinical performance for detecting CIN3 + was inferior to overt cancer, but the scores allowed a moderately accurate estimation.

**Table 1:** Performance of combination with five-miRNAs (miR-126-3p, -451a, -144-3p,-20b-5p and -155-5p) for identifying cervical neoplasia.

	AUC (95% CI)	Sensitivity	Specificity	PLR	NLR	Accuracy	PPV	NPV
Cancer/Normal	0.956 (0.933–0.980)	0.89	0.94	15.43	0.12	0.91	0.97	0.81
SCC/Normal	0.963 (0.940–0.987)	0.91	0.94	15.76	0.10	0.92	0.95	0.88
AD + ADSQ/Normal	0.944 (0.909–0.979)	0.84	0.92	10.48	0.17	0.89	0.86	0.91
CIN3 + /Normal + CIN1	0.836 (0.799–0.873)	0.72	0.82	4.08	0.34	0.75	0.90	0.57

*AUC* area under the curve, *CI* confidence interval, *PLR* positive likelihood ratio, *NLR* negative likelihood ratio. *PPV* positive predictive value, *NPV* negative predictive value, *SCC* squamous cell carcinoma, *ADSQ* adenosquamous carcinoma, *AD* adenocarcinoma.

Five-miRNAs: miR-126-3p ,-451a -144-3p, -20b-5p and -155-5p. The cut-off point was determined by the Youden index. Estimation of AUC: 1.0: perfect match, 1.0–0.9: high accuracy, 0.9–0.7: moderate accuracy, 0.7–0.5: low accuracy, 0.5: chance result.

### Low predicted probability score is associated with a good prognosis

**Figure 2:**
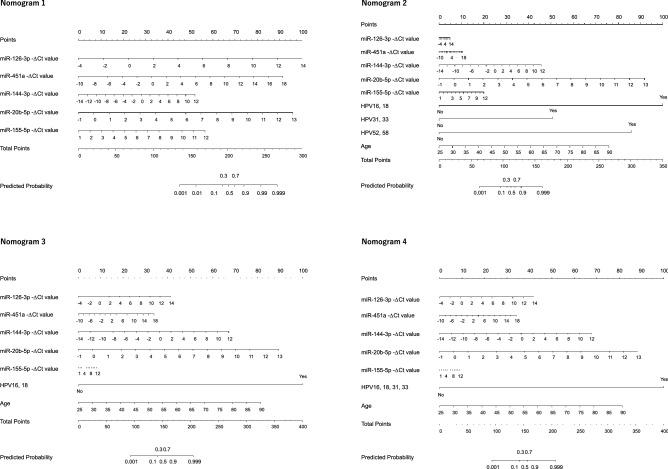
Nomograms predict cervical cancer using patient-specific outcome scores based on summing the individual point total for each −ΔCt value of miRNAs (on the left). After the total points are marked, the outcome score predicted is read. Variables value of Nomogram 1 were constituted by −ΔCt values of 5 miRNAs. Variables value of nomogram 2 by HPV16/18, HPV31/33, HPV52/58 and age in addition to the −ΔCt values of the 5 miRNAs. Variables value of nomogram 3 by HPV16/18 and age in addition to −ΔCt values of the 5 miRNAs. Variables value of nomogram 4 by HPV 16/18/31/33 and age in addition to −ΔCt value of the 5 miRNAs.

**Figure 3:**
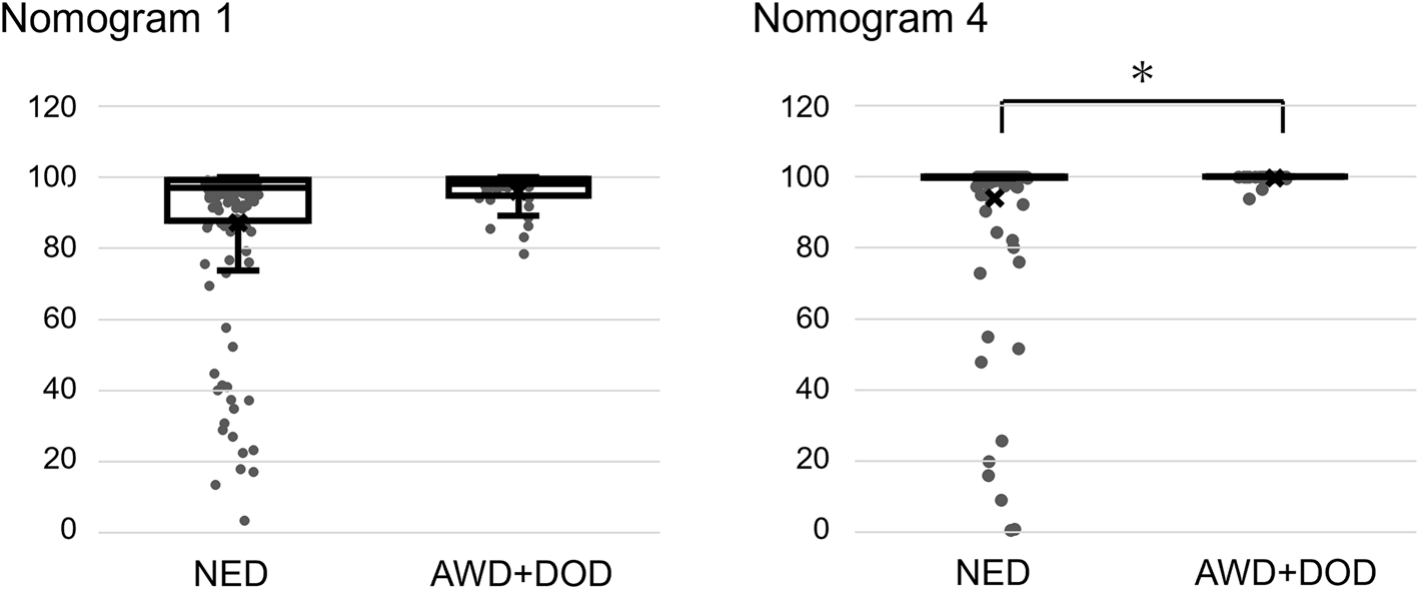
Association between predicted probability and prognosis. Low predicted probability was associated with good prognosis (Nomogram 1: *p* = 0.070, Nomogram 4: *p = 0.005) in patients with cervical cancer by Mann–Whitney U test. NED: no evidence of disease; AWD: alive with disease; DOD: dead of disease. Y-axis indicates predicted probability (%).

We constructed a series of nomograms (Nomogram 1 ~ 4) in Fig. 2. We sought associations between the predicted probability and prognosis in the validation cohort using nomograms. The median predicted probability in the NED and AWD/DOD groups was estimated at 0.971 (0.834–0.910) and 0.981 (0.940–0.980). There was a significant difference between patients with NED and AWD/DOD (Nomogram 4, *p* = 0.005), suggesting that a combination of low expression of miRNAs, age and HPV genotype may predict a favorable prognosis (Fig. 3).

### Predicted probability for cervical cancer in the 2nd set of specimens

In the 2nd set, we examined the predicted probability using the nomograms shown in Fig. 2. Considering nomogram 1 in Supplementary Table S3, 88.6% (39/44) of patients with cervical cancer exhibited a > 83.7% predicted probability. Hence, we selected 83.7% as the cut-off value. None of the normal group reached this cut-off value, suggesting that nomogram 1 was useful for the prediction of cervical cancer. According to the clinical stage classification (FIGO 2018), the positive rate was 72.7% (8/11), 100% (13/13), 86.7% (13/15) and 100% (5/5) of patients at clinical stages I–IV, respectively.

### Nomogram for prediction of cervical cancer and its precursor lesions

Three additional sets of nomograms were constructed to estimate the predicted probability for cervical cancer (Fig. 2) and its precursor lesions (Supplementary Figure S2). Other risk factors including positivity for HPV16, 18, 31, 33, 52 and 58, as well as age, were added to nomogram 2. HPV16, HPV18 and age were added to nomogram 3. Finally, HPV16, 18, 31 and 33, and age, were added to nomogram 4. The cut -off value of CIN3 + and CIN2 + was set at 69.6% and 68.6%, respectively according to the above approach. The value for the predicted probability of each specimen is given in Table 2 showing that the highest accuracy for cancer (0.983), CIN3 + (0.966) and CIN2 + (0.953) was achieved by nomograms 2, 12 and 22, respectively, all of which were calculated by nomogram 2. Thus, nomogram 2 possesses the highest accuracy to predict cancer and its precursor lesions.

**Table 2:** Performance of nomograms for the risk of cervical neoplasia.

	Sensitivity	Specificity	Accuracy	PPV	NPV
**Cancer**					
Nomogram 1	0.886	0.813	0.867	0.951	0.684
Nomogram 2	0.977	1.000	0.983	1.000	0.941
Nomogram 3	0.955	1.000	0.967	1.000	0.889
Nomogram 4	0.955	1.000	0.967	1.000	0.889
**CIN3 + **					
Nomogram 11	0.889	0.813	0.875	0.955	0.619
Nomogram 12	0.958	1.000	0.966	1.000	0.842
Nomogram 13	0.931	1.000	0.943	1.000	0.762
Nomogram 14	0.903	1.000	0.920	1.000	0.696
**CIN2 + **					
Nomogram 21	0.901	0.813	0.888	0.965	0.591
Nomogram 22	0.945	1.000	0.953	1.000	0.762
Nomogram 23	0.879	1.000	0.897	1.000	0.593
Nomogram 24	0.857	1.000	0.879	1.000	0.552

*CIN* cervical intraepithelial neoplasia.

Predicted Probability was calculated using the nomogram in Fig. 3 and Supplementary Figure S2. The cut-off value (Cancer: 83.7%; CIN3 + : 69.6%; CIN2 + : 68.6%) was determined with the data in Supplementary Table S3. More in detail in “materials and methods” section.

### Stable yield of total RNA and predicted probability using specimens left at room temperature

We compared data on the predicted probability assessed using samples stored under different conditions (Supplementary Table S4). The total yield of RNA from samples left overnight at room temperature was 105.6% of that from samples for the same patients frozen immediately after collection. Thus, there was little different yield of RNA after overnight storage at room temperature. Five of 8 cases yielded > cut-off value (83.7%) of predicted probability by nomogram 1regardless of storage conditions. The other 3 cases (ID2, 3 and 5) showed low predicted probability, also in both. Notably, therefore, there were no discrepancies in predicted probability depending on different storage conditions.

## Discussion

Aberrant expression of miRNAs is one of the potential biomarkers for the detection of cervical cancer and its precursor lesions^11,12^. We previously described differences between normal and cervical neoplasia by the fold-change of levels of certain miRNAs. The previous setting was just suited for the comparison between normal and disease groups. Thus, it remained necessary to establish −ΔCt values calculating Ct values of each target relative to the internal reference control to estimate in the external cohort. For the ideal internal reference control, candidate miRNAs would need to be expressed at a constant level in healthy women and through all disease stages. Hence, we preferred to use a novel internal reference control, namely, the average Ct of miR-3180 and miR-7109-5p instead of RNU48 which we previously employed as an internal control (Supplementary Table S1).

In our previous work, we had estimated the accuracy of screening with 4 miRNAs separately, but combining candidate miRNAs offers the possibility of further increasing accuracy^13,19^. Considering that the majority of cervical cancer derives from SCC (80%)^20^, we adopted a group of miRNAs including miR-20b-5p, -126-3p, -451a, and -144-3p, -155-5p for the validation cohort. Although the accuracy of miR-155-5b alone was inferior to the others in Fig. 1b, a combination of the 5 miRNAs showed the minimum AIC value and the maximum AUC compared with other combinations (Supplementary Table S2). This indicated that the combination of 5 miRNAs was best for accurate cervical cancer screening (Table 1).

Nomograms are utilized for predicting the risk of cervical neoplasia or recurrence^21^. MiRNA was one of the variables in a published nomogram with prognostic value for cervical cancer^22^. To the best of our knowledge, there are no published reports regarding the use of nomograms including miRNAs for cervical cancer screening. Hence, we constructed nomograms using 5 miRNAs for cervical cancer screening with the validation cohort and calculated the predicted probability for an external cohort. We also integrated HPV genotypes and patient age into the nomogram. HPV16 and 18 imbued the highest risk for cervical cancer, followed by HPV31 and 33^23,24^. The rate of the predicted probability was consistent with clinical staging. One case (SC-1244 in Supplementary Table S3 and S4) at stage IIIC1r showed low predicted probability in nomograms 1, 3 and 4. In this patient, we noticed necrosis on the surface of the cervix during metastasis assessment by colposcopy (Supplementary Figure S3). This condition could have resulted in our inability to acquire a suitable specimen for miRNA analysis. Another case (SC-1170) at stage IA1 had only a 2 mm depth stromal invasion. The low score of predicted probability in cervical cancer was associated with good prognosis during the 2-year observation period (Fig. 3), and a low score was consistent with early clinical stage. Despite some analyses linking the aberrant expression of a combination of miR-216b, -585b and -7641^25^ and others^22^ with significant differences of patient survival, there are no reports regarding the association between prognosis and the expression of the miRNAs described in the present paper.

In the clinical setting, it may happen that collected samples are left at room temperature for some time. It would be practically important to know that the results of predicted probability analyses would still be reliable when using such material rather than specimens frozen immediately after acquisition. As shown in Supplementary Table S4, we found no difference in the yield of RNA irrespective of storage conditions and importantly, there was no difference in predicted probability estimates. Thus, it might not be necessary to freeze specimens immediately on collection. This is an advantage of a screening test using miRNAs from cervical mucus. Therefore, specimens derived from liquid-based cytology^26^ or self-sampling^27^ may be an alternative approach in future.

This paper has some limitation. The samples were collected in a single University hospital. Therefore, cohorts may differ from the general population. Conversely, characteristics of each specimen were analyzed in more detail including histology and HPV genotyping (Supplementary Table S5). Compared to our previous report, the performance of the ROC with the novel internal control reference was little different from our previous report. However, the novelty of the present paper is to show the predicted probability for patients with cervical cancer and its precursor lesions in an external cohort. As we know that persistent high-risk HPV infections worsen the risk of cervical cancer, nomograms including HPV genotypes could be more promising for predicting full-blown cervical cancer in individuals with precursor lesions. We showed that miRNAs from mucus are stable when the samples were left at room temperature overnight. This is approximately 20–25 ℃ throughout the year, but we have not tested higher temperatures that might apply elsewhere, and we also did not test a longer period of time. Stability of miRNAs was reported to be dependent on their specific sequence, storage or freeze–thaw conditions of either sera or plasma^28–32^. More information regarding the different conditions for storage of larger numbers of specimens could address this.

In summary, here we identified miRNAs highly expressed in cervical mucus of women with a range of lesions from normal through to cervical neoplasms. A combination of 5 miRNAs and an internal reference control was best suited for use a diagnostic marker of cervical neoplasia. Highly reliable performance of miRNA prediction was shown using several different nomograms on the external cohort. We further showed that the predicted probability calculated from analysing specimens either immediately frozen or left at room temperature overnight was identical. We conclude that the development of tests to detect aberrant expression of miRNAs could offer useful candidate ancillary screens for cervical neoplasia in a clinical setting.

## Materials and methods

### Preparation of the study subjects

**Figure 4:**
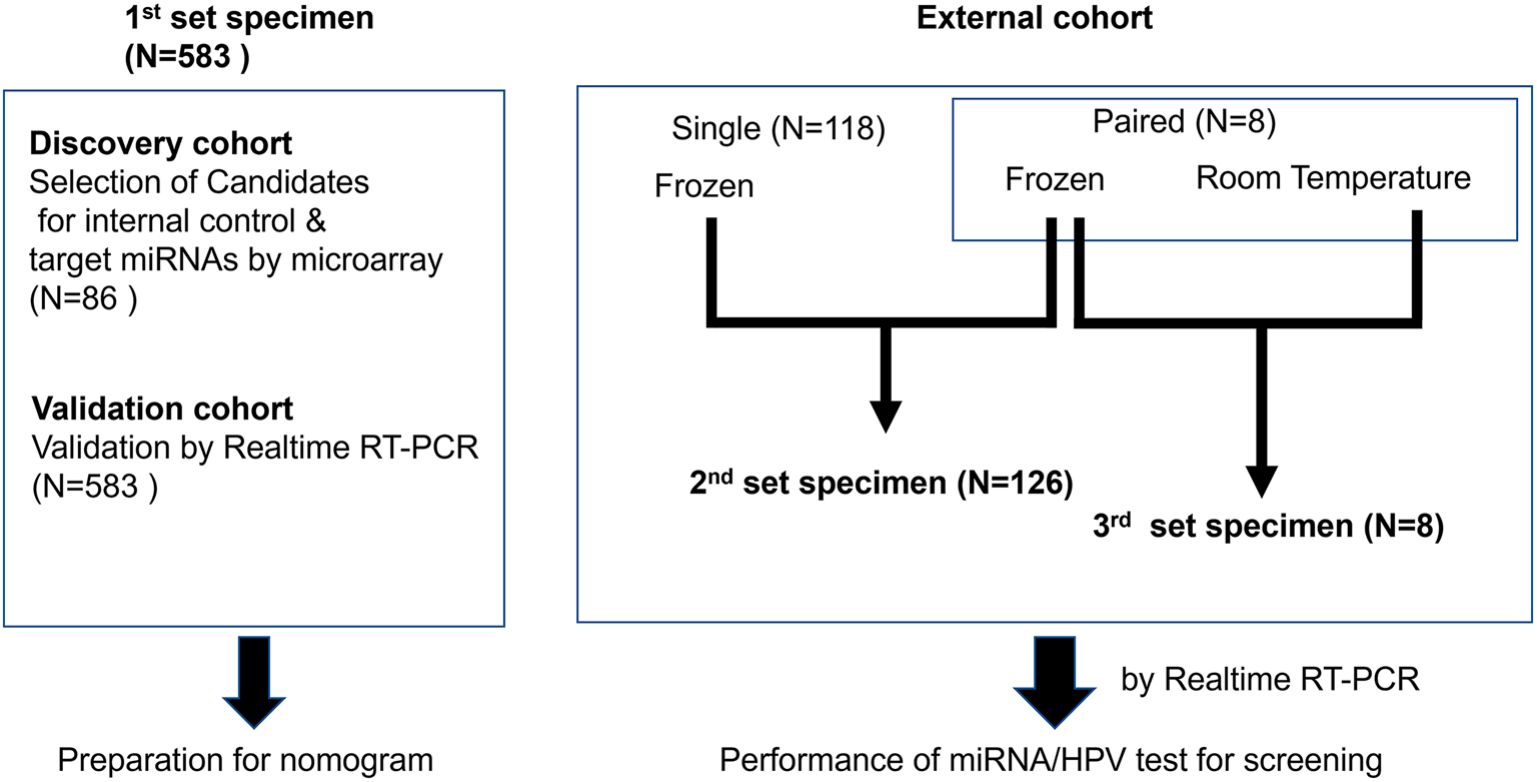
Outline of the experimental design indicating associations between specimen sets and the following analysis.

We performed a series of experiments to compare the level of miRNAs with age, histology and HPV genotype. Estimating the performance of a biomarker depends on the components of the disease category in the enrolled patients. We prepared three sets of specimens, as shown in Fig. 4. The 1st set was taken from a total of 583 individuals including those with cervical neoplasia and normal controls (Supplementary Table S5). As a discovery cohort, a total of 86 of these specimens was used to select the candidate targets or reference miRNAs by microarray analysis (Toray, Kamakura, Japan)^15^. For the validation cohort, the whole cohort of 583 specimens was used to investigate the performance of the level of miRNAs for predicting cervical neoplasia following the construction of a nomogram for that purpose. The external cohort (Supplementary Table S3) was then examined using the estimation of the predicted probability with a 2nd and 3rd set of specimens. Sample of cervical mucus from the uterine cervix were collected using a 1 cm-diameter cotton swab. The specimens from the 1st and 2nd set were immediately stored at − 80 °C following collection. The split specimens from each of 8 patients with cervical cancer were taken for the 3rd set. One of these was immediately frozen, whereas the other was left at room temperature overnight and only stored at − 80 °C on the following day. At the same time as the mucus samples were taken, a cervical brush was inserted into the cervical canal to collect both ectocervical and endocervical cells for HPV genotyping. This material was also stored at − 80 °C. The median age of the consecutive patients was 39 years (range 21–89 years) in the 1st set, 42.5 years (range 22–94) in the 2nd set and 50 years (range 38–68) in the 3rd set.

Patients attended the outpatient clinic at Fujita Health University Hospital, Aichi prefecture, Japan, for routine gynecological examinations between October 2014 and September 2021. Exclusion criteria for patients were described previously^15^. The study protocol was approved by the Ethics Committees of Fujita Health University and the National Institute of Infectious Diseases. Written informed consent was obtained from each patient. All procedures were performed in accordance with the approved guidelines and regulations.

### Real-time RT-PCR

Total RNA was extracted from the cotton swabs using miRNeasy Mini Kits (QIAGEN GmbH, Hilden, Germany). For the 1st set, the candidate miRNAs were validated by real-time RT-PCR. MiRNAs were quantified using the following TaqMan™ MicroRNA Assays: hsa-miR-20b-5p, 001014; hsa-miR-126-3p, 002228; hsa-miR-451a, 001141; hsa-miR-144-3p, 002676; hsa-miR-155-5p, 002623; hsa-miR-3180, 463043_mat; hsa-miR-7109-5p, 466424_mat; and RNU48 small nucleolar RNA, 001006, all from Thermo Fisher Scientific, Waltham, MA, USA. Conditions for quantitative real-time PCR were as previously reported^15^. MiRNA levels were normalized against the combination of the average signal of both miR-3180 and miR-7109-5p and presented as −ΔCt values.

### HPV genotyping

HPV genotyping was performed by PCR with PGMY primers, followed by reverse line blot hybridization^33^. This assay can detect the following 31 HPV genotypes: HPV 6, 11, 16, 18, 26, 31, 33, 34, 35, 39, 40, 42, 44, 45, 51, 52, 53, 54, 55, 56, 57, 58, 59, 66, 68, 69, 70, 73, 82, 83 and 84.

### Nomogram for prediction of cervical cancer and its precursor lesions

Nomograms were formulated to provide visualized risk prediction for cervical neoplasia based on the results of multivariable analyses using free open-source *R* statistical software 4.0.3 (www.r-project.org) “rms” package. First, we used the levels of 5 miRNAs (-ΔCt values of miR-126-3p, -451a, -144-3p, -20b-5p, -155-5p) as variables in nomogram 1 (Fig. 2). According to the nomogram, patients with 135.6, 201.1 and 266.7 total points corresponded to 0.1%, 50% and 99.9% of predicted probability for cervical cancer, respectively. We adopted other variables including age and HPV genotype in addition to the levels of miRNAs, as shown in Supplementary Figure S2.

### Statistical analysis

Comparisons between data points were undertaken using NormFinder (version 0953; https://moma.dk/normfinder-software), an algorithm for identifying the optimal normalization gene among a set of candidates^34^. Exponentially transformed Ct values (2^−Ct^) were used as input data and genes were ranked based on stable expression in a particular sample set using a specific design of experiments^35^. The lowest value calculated by the software indicates the most stable internal expressed gene for optimal normalization. Statistical analyses were performed using SPSS for Windows (ver. 22.0.0.0; IBM Corp, Armonk, NY, USA). The Jonckheere–Terpstra trend test and Kruskal–Wallis one-way analysis of variance were adopted to compare overall differences among disease categories. We compared the fold-difference of all groups using two-tailed Mann–Whitney U tests with Bonferroni correction. A receiver operating characteristic (ROC) curve analysis was performed, and the area under the ROC curve (AUC) was calculated to evaluate the diagnostic value. We unified different 5 miRNAs as markers for use in the determination of predictive probability through logistic regression and then constructed ROC curves according to the probability. We used Akaike information criterion (AIC) to compare the goodness-of-fit of each model of combined miRNAs^36^. For prognosis, enrolled patients with cancer in the 1st (N = 146) and 2nd (N = 23) set were classified into no evidence of disease (NED) (N = 138) and alive with disease (AWD)/dead of disease (DOD) (N = 31) during the observation period (median 2 years). Statistical analysis of the predicted probability differences between two groups was determined by Mann–Whitney U test.

## Supplementary Information

Supplementary Information 1.

Supplementary Information 2.

Supplementary Information 3.

Supplementary Information 4.

Supplementary Information 5.

Supplementary Information 6.

Supplementary Information 7.

Supplementary Information 8.
