# Supplementary material for: Nomogram for predicted probability of cervical cancer and its precursor lesions using miRNA in cervical mucus, HPV genotype and age
Source: Sci Rep. 2022 Sep 28;12:16231. doi: 10.1038/s41598-022-19722-3 (PMC9519568; doi:10.1038/s41598-022-19722-3)
Supplement: Supplementary file 1 — Supplementary Information 1. [file 41598_2022_19722_MOESM1_ESM.docx]

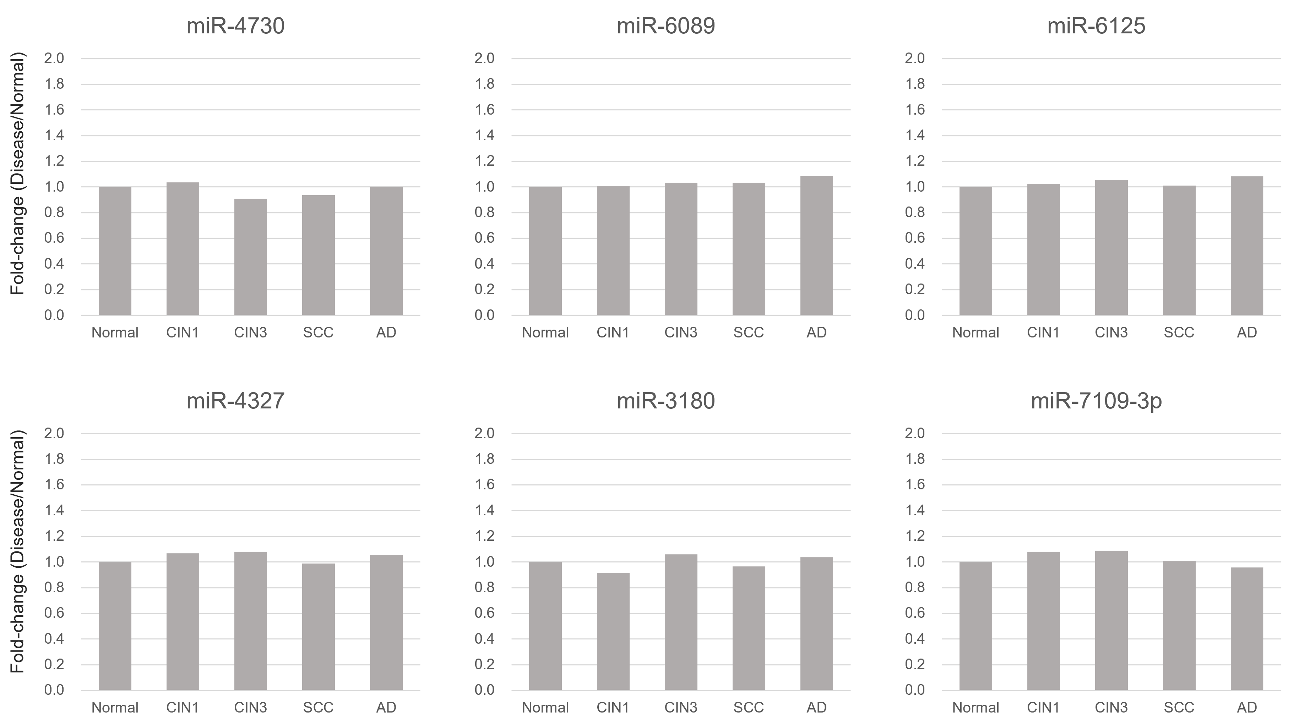


Figure S1 Candidate miRNAs for internal control reference controls between normal and cervical neoplasia by microarray analysis. Equivalent fold changes of expression levels of miRNAs between normal and cervical neoplasia were shown. Details described in materials and methods. CIN: cervical intraepithelial neoplasia, SCC: squamous cell carcinoma, AD: adenocarcinoma.
