# Supplementary material for: Nomogram for predicted probability of cervical cancer and its precursor lesions using miRNA in cervical mucus, HPV genotype and age
Source: Sci Rep. 2022 Sep 28;12:16231. doi: 10.1038/s41598-022-19722-3 (PMC9519568; doi:10.1038/s41598-022-19722-3)
Supplement: Supplementary file 2 — Supplementary Information 2. [file 41598_2022_19722_MOESM2_ESM.docx]

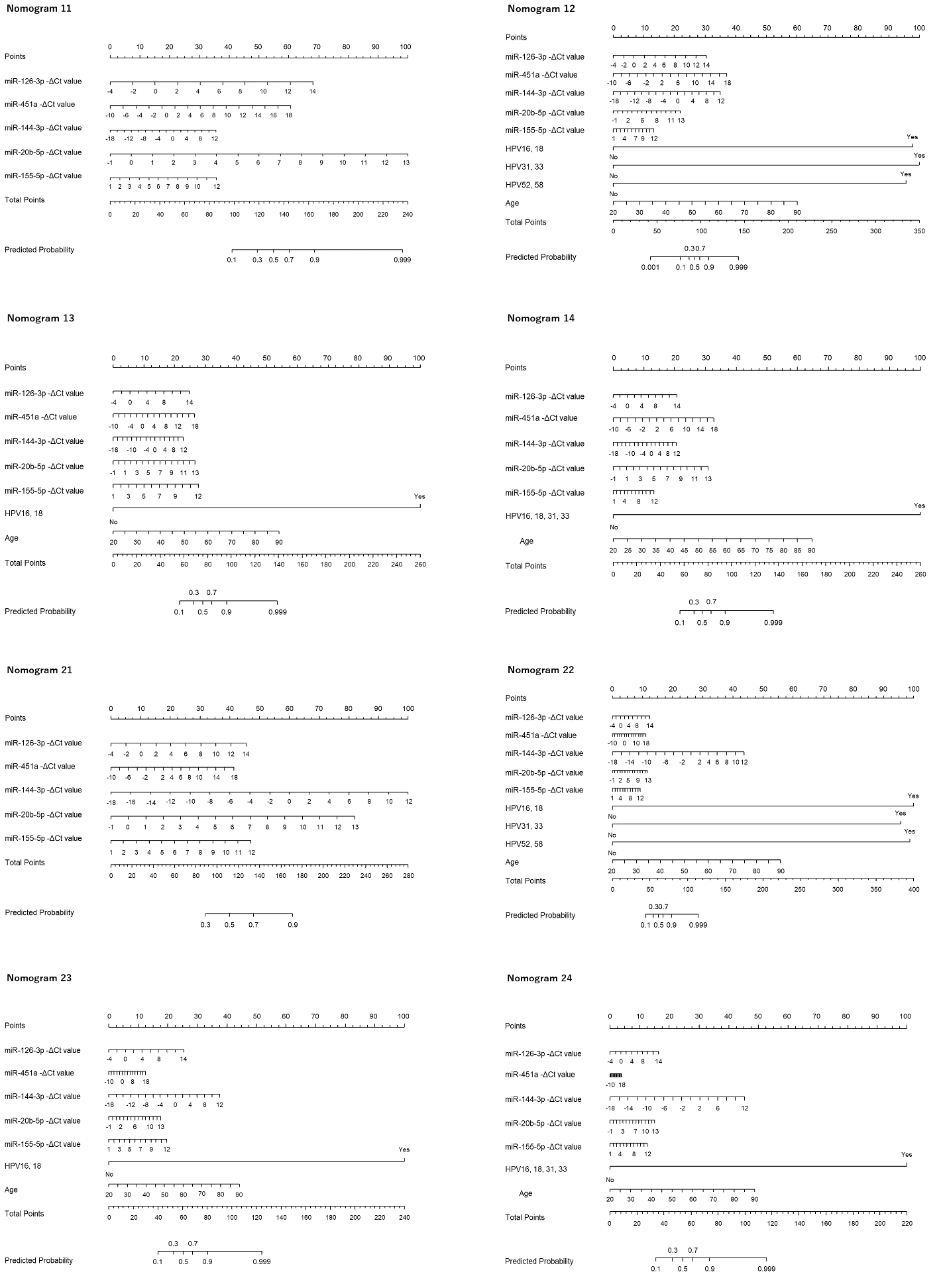


Figure S2

Nomograms predict cervical cancer and its precursor lesions. Nomogram 11-14 was constructed for CIN3 and worse, and nomogram21-24 for CIN2 and worse.  Variables value of Nomogram 11 and 21 was constituted by -ΔCt value of five miRNAs. Variable values of nomogram 12 and 22 by HPV16/18, HPV31/33, HPV52/58 in addition to  -ΔCt value of five miRNAs. Variable values of nomogram 13 and 23 by  HPV16/18 in addition to  -ΔCt value of five miRNAs. Variable values of nomogram 14 and 24 by HPV 16/18/31/33 in addition to  -ΔCt value of five miRNAs.
