# Supplementary figures and images for: Nomogram for predicted probability of cervical cancer and its precursor lesions using miRNA in cervical mucus, HPV genotype and age
Source: Sci Rep. 2022 Sep 28;12:16231. doi: 10.1038/s41598-022-19722-3 (PMC9519568; doi:10.1038/s41598-022-19722-3)

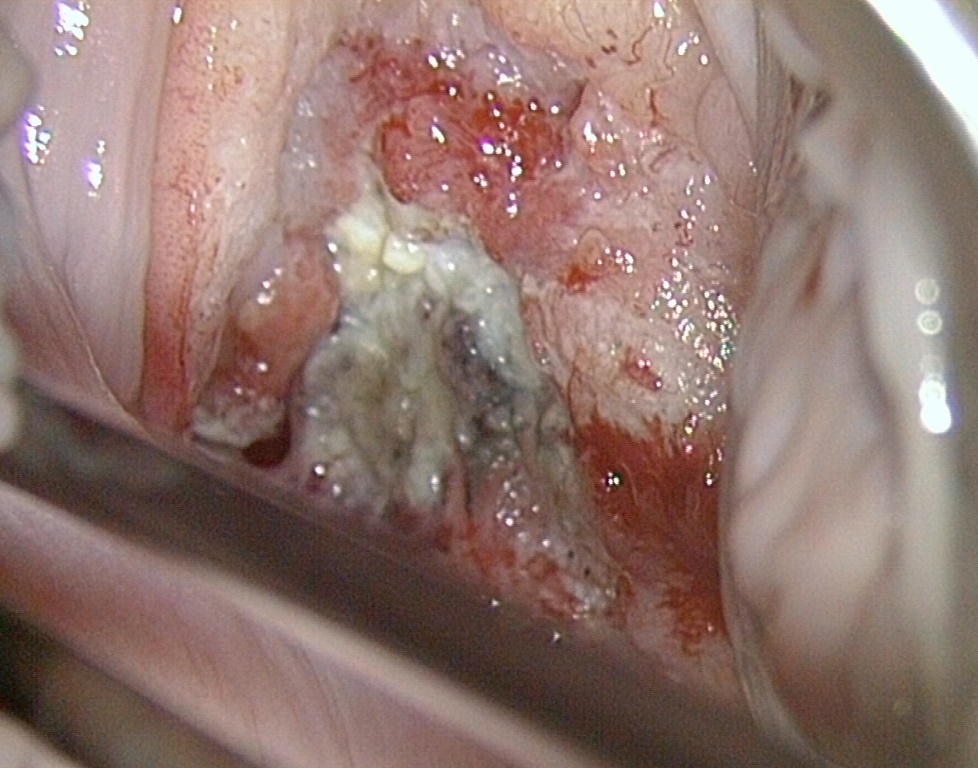


Figure S3

Colposcopy of the ID 6 in Table S5. Necrotic tissue is visible on the cervix.

Supplement: Supplementary file 3 — Supplementary Information 3. [file 41598_2022_19722_MOESM3_ESM.docx]
