# Supplementary material for: Nomogram for predicted probability of cervical cancer and its precursor lesions using miRNA in cervical mucus, HPV genotype and age
Source: Sci Rep. 2022 Sep 28;12:16231. doi: 10.1038/s41598-022-19722-3 (PMC9519568; doi:10.1038/s41598-022-19722-3)
Supplement: Supplementary file 4 — Supplementary Information 4. [file 41598_2022_19722_MOESM4_ESM.docx]

Table S1 Association between mean Ct values and patient group

|  | Normal | Cervical neoplasia | Mann–Whitney U *p* value | NormFinder Stability value |
| --- | --- | --- | --- | --- |
| RNU48 | 24.7±1.7 | 24.2±1.6 | 0.012 | 0.209 |
| miR-3180 | 33.8±0.9 | 33.8±1 | 0.910 | 0.075 |
| miR-7109-5p | 31.3±1.4 | 31.4±1.3 | 0.383 | 0.116 |
| Average Ct of miR-3180  and miR-7109-5p | 32.5±1 | 32.6±0.9 | 0.712 | 0.069 |
| *Footnote*: Ct values were shown as mean ± standard deviation (SD), Stability value: High expression stability is indicated by a low stability value, *p* value <0.05 as significant | | | | |
