# Supplementary material for: Nomogram for predicted probability of cervical cancer and its precursor lesions using miRNA in cervical mucus, HPV genotype and age
Source: Sci Rep. 2022 Sep 28;12:16231. doi: 10.1038/s41598-022-19722-3 (PMC9519568; doi:10.1038/s41598-022-19722-3)
Supplement: Supplementary file 5 — Supplementary Information 5. [file 41598_2022_19722_MOESM5_ESM.docx]

Table S2 Akaike Information Criterion analysis of miR-126-3p, -451a, 144-3p, -20b-5p and -155-5p and their variable combinations in patients with cervical cancer

|  |  | Normal vs Cancer | |
| --- | --- | --- | --- |
| Combination with miRs |  | AIC | AUC (95% CI) |
| -126-3p ,-451a ,-144-3p ,-20b-5p ,-155-5p |  | 136.9 | 0.956 (0.933 - 0.980) |
| -126-3p ,-451a ,-144-3p ,-20b-5p |  | 146.1 | 0.952 (0.927 - 0.977) |
| -126-3p ,-451a ,-144-3p ,-155-5p |  | 141.9 | 0.950 (0.923 - 0.977) |
| -126-3p ,-451a ,-20b-5p ,-155-5p |  | 137.4 | 0.956 (0.933 - 0.979) |
| -126-3p ,-144-3p ,-20b-5p ,-155-5p |  | 140.2 | 0.953 (0.928 - 0.978) |
| -451a ,-144-3p ,-20b-5p ,-155-5p |  | 141.0 | 0.953 (0.928 - 0.978) |
| *Footnote*: AIC: Akaike Information Criterion analysis, AUC: area under the curve, CI: confidence interval. AIC was calculated by patients with cancer (N=168) and normal (N=87) in the validation cohort. | | | |
