# Supplementary material for: Nomogram for predicted probability of cervical cancer and its precursor lesions using miRNA in cervical mucus, HPV genotype and age
Source: Sci Rep. 2022 Sep 28;12:16231. doi: 10.1038/s41598-022-19722-3 (PMC9519568; doi:10.1038/s41598-022-19722-3)
Supplement: Supplementary file 7 — Supplementary Information 7. [file 41598_2022_19722_MOESM7_ESM.docx]

Table S4 Association between yield of total RNA and predicted probability in different storage conditions

| Patient ID | ID | | Total RNA yield (µg) | | | Predicted Probability (%) | |  |
| --- | --- | --- | --- | --- | --- | --- | --- | --- |
|  | Immediate frozen | Left at room temperature | Immediate frozen | Left at room temperature | Left/Immediate frozen | Immediate frozen | Left at room temperature |  |
|  |  |  |  |  |  |  |  |  |
| 1 | Sc-1129 | Sc-1130 | 23.7 | 19.4 | 81.8% | 94.8 | 99.3 |  |
| 2 | Sc-1168 | Sc-1169 | 5.9 | 12.9 | 221.0% | 3.6 | 0.5 |  |
| 3 | Sc-1170 | Sc-1171 | 19.5 | 25.2 | 129.1% | 3.4 | 0.4 |  |
| 4 | Sc-1179 | Sc-1180 | 27.2 | 22.4 | 82.5% | 97.8 | 89.8 |  |
| 5 | Sc-1244 | Sc-1245 | 18.2 | 20.2 | 111.2% | 38.1 | 11.9 |  |
| 6 | Sc-1253 | Sc-1252 | 10.9 | 17 | 156.2% | 99.5 | 93.5 |  |
| 7 | Sc-1263 | Sc-1264 | 6.4 | 5.3 | 82.6% | 99.9 | 99.9 |  |
| 8 | Sc-1269 | Sc-1270 | 27.5 | 27.4 | 99.9% | 99.4 | 99.5 |  |
| Median |  |  | 18.9 | 19.8 | 105.6% | 96.3 | 91.7 |  |
| *Foot note* : Predicted Probability was calculated using the nomogram 1 in Figure 2. | | | | | | | |  |
